# The Birthplace of Modern Dentistry

**Published:** 1873-12

**Authors:** 


### EDITORIAL, ETC.

*The Birthplace of Modern Dentistry.*—In a recent publication entitled "The Monumental City," edited by G. W. Howard, we find the following:

"In one sense Dentistry is a modern science. That it was known and practiced in ancient Egypt, is evident from the discoveries in the Pyramids; and that a measure of skill was attained, is apparent from the remnants of workmanship still preserved, but its disappearance was as complete as the passing away of the Lost Arts, and in the eighteenth century practical Dentistry had no existence. During that period public attention was attracted to the subject, and a number of theoretical treatises were written by enthusiastic physicians. It was not however until the early part of the present century, that the views of its votaries assumed a practical direction sufficient to elevate it into a distinct science. America had the honor of nursing it through its infant struggles, and Baltimore may with propriety be called the birthplace of modern Dentistry.

"In 1826, the "Principles of Dental Surgery" appeared in London, a work written by Leonard Koecker, a Baltimore physician. This was followed, in 1839, by the establishment in Baltimore of the *American Journal of Dental Science*, and in 1840 the 'Baltimore College of Dental Surgery' was organized under a charter granted by the Legislature of Maryland. Shorly afterwards the great standard work of Dr. Chapin A. Harris, the 'Principles and Practice of Dental Surgery,' made its appearance. The Baltimore College of Dental Surgery, in which Dr. Harris was for a number of years a leading Professor, is not only teh oldest, but if we may judge by results, one of the best in the world. Seven hundred and nine students have been graduated by this institution since its foundation. They are distributed pretty generally through the civilized portion of the globe, and wherever they have located the fame of their *Alma Mater* has accompanied them. Nearly every Dental College in this country contains in its faculty some graduate of this institution, and a large majority of the Court Dentists of Europe acknowledge their obligations to the same source. The Museum of the College is, without doubt, among the most complete in the United States, possessing a large and rare collection of pathological specimens, while the course marked out for the students is very comprehensive, embracing anatomy, physiology and chemistry, and materia medica the lectures upon these subjects being very full and minute.

Baltimore took the lead in this department of science from the first, and has steadily maintained her position. The students matriculating in this city are not confined to the United States, but many come from the enlightened centres of Europe to avail themselves of the advantages which Baltimore extends to those desirous of acquiring a knowledge of Dentistry."

